# Real-world practices of low-molecular-weight heparin for venous thromboembolism prophylaxis in patients hospitalized with COVID-19: a multicenter prospective study from China

**DOI:** 10.1186/s12959-025-00741-9

**Published:** 2025-06-20

**Authors:** Feiya Xu, Yuzhi Tao, Lijun Chen, Yunhui Zhang, Binliang Wang, Jing Han, Chaosheng Deng, Weijia Liu, Guohui Fan, Rui Liang, Zhaofei Chen, Yinong Chen, Kaiyuan Zhen, Yunxia Zhang, Zhu Zhang, Shuai Zhang, Jun Wan, Wanmu Xie, Peiran Yang, YuTing Kang, Dingyi Wang, Chen Wang, Zhenguo Zhai

**Affiliations:** 1 Institute of Clinical Medical Sciences), China-Japan Friendship Hospital, Chinese Academy of Medical Sciences & Peking Union Medical College, Beijing, 100005 China; 2 The First Bethune Hospital of Jilin University, Jilin University, Changchun, 130021 China; 3 National Center for Respiratory Medicine, State Key Laboratory of Respiratory Health and Multimorbidity, National Clinical Research Center for Respiratory Diseases, Institute of Respiratory Medicine, Department of Pulmonary and Critical Care Medicine, Center of Respiratory Medicine, Chinese Academy of Medical Sciences, China-Japan Friendship Hospital, Beijing, 100029 China; 4 Department of Pulmonary and Critical Care Medicine, The First People’s Hospital of Yinchuan, Yinchuan, 750001 China; 5 Department of Pulmonary and Critical Care Medicine, First People’s Hospital of Yunnan Province, Yunnan, 650034 China; 6 Department of Pulmonary and Critical Care Medicine, Taizhou First People’s Hospital, Taizhou, 318020 China; 7 Department of Pulmonary and Critical Care Medicine, Guizhou Provincial People’s Hospital, Guiyang, 550000 China; 8 Department of Pulmonary and Critical Care Medicine, The First Affiliated Hospital of Fujian Medical University, Fuzhou, 350000 China; 9 Beijing University of Chinese Medicine China-Japan Friendship School of Clinical Medicine, Beijing, 100105 China; 10 Peking University China-Japan Friendship School of Clinical Medicine, Beijing, 10029 China; 11 Department of Pulmonary and Critical Care Medicine, Beijing Anzhen Hospital, Capital Medical University, Beijing, 100029 China; 12 Department of Physiology, State Key Laboratory of Medical Molecular Biology, Institute of Basic Medical Sciences, School of Basic Medicine Peking Union Medical College, Chinese Academy of Medical Sciences, Beijing, 100005 China; 13 Department of Science Research, Beijing Hospital, National Centre of Gerontology, Institute of Geriatric Medicine, Chinese Academy of Medical Sciences, Beijing, China

**Keywords:** Thromboprophylaxis, Low-molecular-weight heparin, Venous thromboembolism, COVID-19

## Abstract

**Background:**

Effective thromboprophylaxis is critical to reducing mortality and improving clinical outcomes in COVID-19 patients. Despite guidelines recommending prophylactic anticoagulation, particularly for those in intensive care, real-world adherence and optimal venous thromboembolism (VTE) prevention strategies remain challenging, particularly in populations with complex comorbidities.

**Methods:**

A prospective study was conducted on patients hospitalized with moderate, severe, and critical COVID-19 in six Chinese hospitals during the Omicron pandemic (December 2022-January 2023). The dose and duration of low-molecular-weight heparin (LMWH) were recorded. VTE, all-cause mortality and major bleeding events during hospitalization and 90-days follow-up were analyzed as endpoints.

**Results:**

Among 4,236 COVID-19 patients, 1575 (37.09%) received LMWH prophylaxis, with 592 (37.6%) receiving reduced dosage (< 4000IU/24 h). The multivariable logistic regression model revealed that age ≥ 65, elevated D-dimer levels, severely ill at admission and concomitant use of antiviral drugs or corticosteroids were the main factors influencing the initiation of LMWH thromboprophylaxis in hospitalized COVID-19 patients. Patients who were critically ill at admission were more likely to receive reduced doses of LMWH. The duration of thromboprophylaxis over 7 days was associated with reduced estimated glomerular filtration rate (eGFR) and concomitant use of antiviral drugs or corticosteroid, whereas shorter durations were observed in patients with platelet less than 100*10^9^/L and anemia.

**Conclusion:**

Real-world thromboprophylaxis in hospitalized COVID-19 patients vary widely, with a significant proportion receiving lower-than-conventional doses of LMWH. There is a need for individualized thromboprophylaxis strategies that consider patient-specific factors such as disease severity, renal function, low platelet and anemia to optimize outcomes.

**Supplementary Information:**

The online version contains supplementary material available at 10.1186/s12959-025-00741-9.

## Introduction

The COVID-19 has infected millions of people worldwide and become a major public health threat [1, 2]. SARS-CoV-2 infection induces endothelial dysfunction, platelet activation, and inflammatory responses [3–5], leading to a prothrombotic state and an elevated risk of venous thromboembolism (VTE) in COVID-19 patients [6, 7]. Furthermore, the presence of VTE in COVID-19 patients is associated with increased mortality and poor clinical outcomes [8, 9], underscoring the critical need for effective thromboprophylaxis in this population. Emerging research indicates that anticoagulation should be considered at all stages of COVID-19 treatment [10].

Current guidelines recommend the use of prophylactic doses of anticoagulants for COVID-19 patients admitted to the intensive care unit (ICU), while suggesting therapeutic doses for those in non-ICU settings [11–13]. However, adhering to guidelines in clinical practice is challenging due to the high risk of bleeding associated with anticoagulation [14], and the need to adjust for complex patient-specific conditions. Previous research has shown a lower rate of VTE prophylaxis among Chinese in-hospital patients compared to international counterparts [15]. This discrepancy highlights the need for a comprehensive assessment of the current state of VTE prevention in in-hospital COVID-19 patients.

Understanding the reasons for the gap in guideline adherence is essential to improve thromboprophylaxis and reduce both disease and socioeconomic burden. Limited evidence of anticoagulation strategies in fragile populations complicates the choice of appropriate dosage and duration of thromboprophylaxis in COVID-19 patients. Since low-molecular-weight heparins (LMWHs) are the first-line anticoagulants recommended by guidelines for in-hospital thromboprophylaxis of COVID-19 [12, 16, 17], this study aimed to investigate real-world pharmacological thromboprophylaxis strategies in hospitalized COVID-19 patients in China.

## Method

### Study design

Patients hospitalized with COVID-19 were consecutively enrolled in six general hospitals between December 2022 and January 2023 during the Omicron pandemic in China. The geographical distribution of enrolled patients was shown in supplementary Table 1. Inclusion criteria were age over 18 years; having a nucleic acid amplification test positive for SARS-CoV-2 and length of hospitalization for more than 24 h. Exclusion criteria were pregnant; COVID-19 without pneumonia; having incomplete detailed data on anticoagulants; or receiving therapeutic anticoagulation for at least 1 month before SARS-CoV-2 infection. The patients enrolled received regular follow-ups during the 90 days after discharge, either through clinic or phone visits.

### Data collection

Demographics, comorbidities, prescriptions, other laboratory data and follow-up information were collected using an electronic reporting form (eCRF). The physicians at each institution were responsible for data entry into the eCRF. In addition, data were manually checked for missing or contradictory inputs and values outside the expected ranges at the research-based office.

#### Definitions

The illness severity of COVID-19 was defined according to the diagnosis and treatment protocol for novel coronavirus pneumonia (version 10) [18], published by the National Health Commission of China as follows: [1] mild: the clinical symptoms are mild, and there was no sign of pneumonia on chest imaging; [2] moderate: fever and respiratory symptoms, with evidence of pneumonia on radiologic imaging; [3] severe: any of the following symptoms and signs: respiratory distress with respiratory rate ≥ 30 breaths/min, SpO_2_ ≤ 93% at rest, PaO_2_/FiO_2_ ≤ 300 mmHg (1 mmHg = 0.133 kPa); [4] critical: any of the following conditions: respiratory failure requiring mechanical ventilation, shock, or other organ failure requiring admission to the ICU. Prophylactic anticoagulation was defined as VTE prophylaxis with LMWHs for more than 3 days. Reduced prophylactic dose refers to LMWH dosage less than 4000IU per 12 h.

### Clinical outcomes

The primary clinical outcomes were symptomatic VTE, including deep vein thrombosis (DVT) and pulmonary embolism (PE) confirmed by imaging examinations (ultrasound, contrast-enhanced computed tomography, ventilation-perfusion lung scintigraphy, pulmonary angiography, or contrast venography), and all-cause mortality during hospitalization and follow-up. All imaging interpretations were independently verified by two radiologists, with discrepancies resolved by a third expert. The secondary outcome was major bleeding (MR) [19] and clinically relevant non-major bleeding (CRNMB) [20]. MR consisted of a reduction in the hemoglobin level by at least 2 g/dL, transfusion of at least 2 U of blood, or symptomatic bleeding in a critical area or organ according to the International Society of Thrombosis and Hemostasis (ISTH). CRNMB consisted of any sign or symptom of hemorrhage (e.g., more bleeding than would be expected for a clinical circumstance, including bleeding found by imaging alone) that does not fit the criteria for the ISTH definition of major bleeding but does meet at least one of the following criteria: (i) requiring medical intervention by a healthcare professional; (ii) leading to hospitalization or increased level of care; (iii) prompting a face to face (i.e., not just a telephone or electronic communication) evaluation.

### Statistical analysis

Categorical variables are presented as numbers and percentages, while continuous variables are presented as the mean ± SD or median (IQR) based on their distributions. Categorical variables were compared using the chi-square or Fisher’s exact test as appropriate. Continuous variables were analyzed with Student’s t-test or Wilcoxon rank-sum test based on distribution. Missing data were checked for patterns and handled by complete-case analysis when the proportion was low. For variables with more missing values, multiple imputations were applied as appropriate. Outliers were identified using range checks based on clinical knowledge, and any values found to be implausible were either corrected or excluded if verification was not possible. To identify the clinical characteristics associated with the implementation of pharmacological thromboprophylaxis and reduced-dosage prophylaxis, the adjusted odds ratio (OR) and their 95% confidence intervals (CIs) were estimated using multivariable logistic regression model. We calculated the crude/adjusted ORs considering several confounding factors. Statistical significance was set at *P* < 0.05. All analyses were performed using SAS (SAS 9.4, SAS Institute Inc.).

## Results

### Baseline characteristics and clinical outcomes of in-hospital COVID-19 patients with and without LMWH thromboprophylaxis

**Fig. 1:**
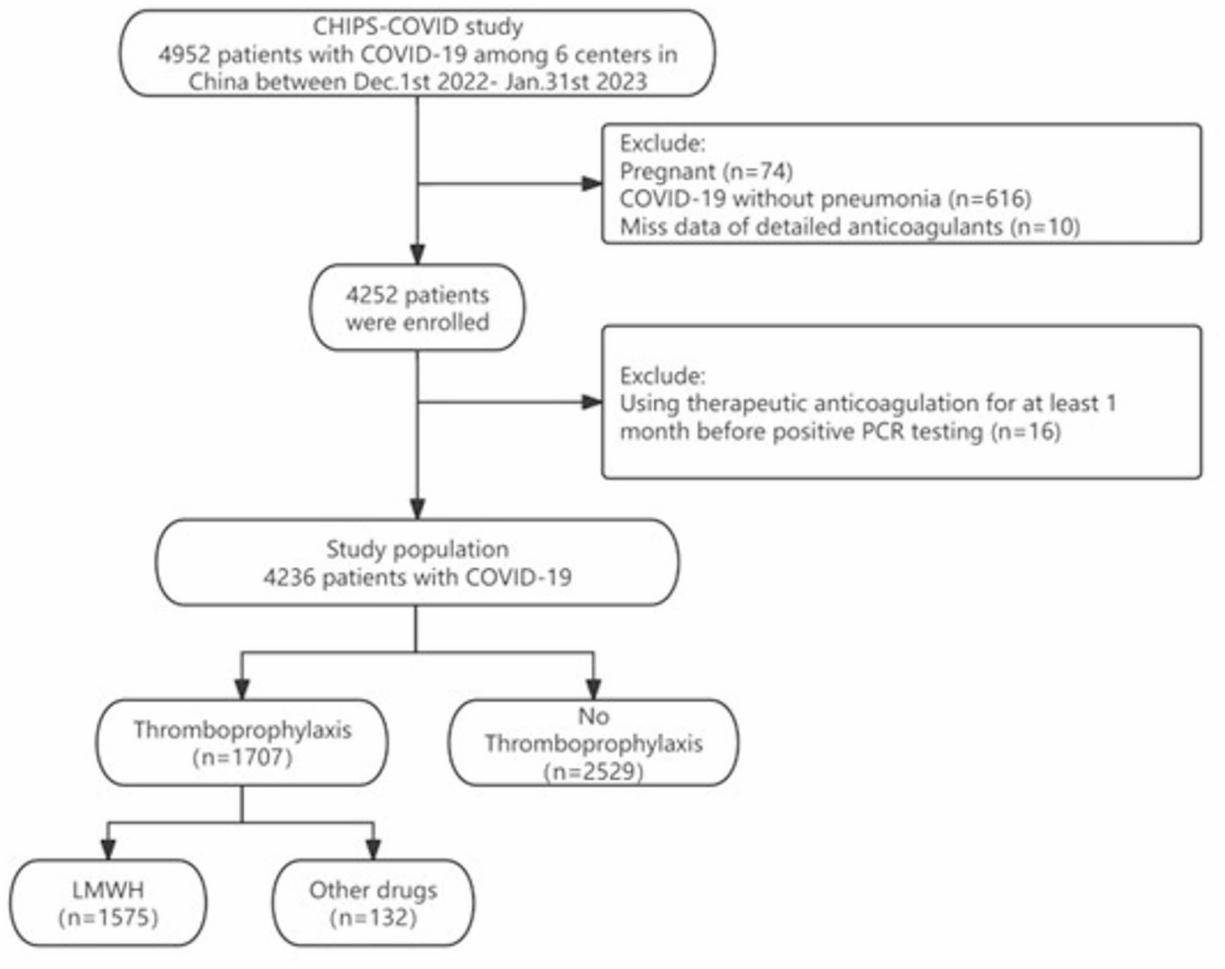
Flowchart Note: Flow chart on the selection process. Patients hospitalized with COVID-19 included between December 2022 and January 2023. PCR, Polymerase Chain Reaction; LMWH, low-molecular-weight heparin

Of 4952 eligible patients, 4236 patients were included in the full analysis set, with 1,575 (37.09%) receiving LMWH prophylaxis (Fig. 1). Additionally, 52 patients received unfractionated heparin and 310 patients received oral anticoagulants, as shown in supplementary Table 2. Patients who received LMWH prophylaxis were older, predominantly male (65.50% vs. 57.73%, *P* < 0.001), had higher baseline D-dimer levels, and more severe conditions at admission, along with higher VTE risk scores and more cardiovascular comorbidities (77.30% vs. 73.22%, *P* = 0.0077). In terms of clinical outcomes, patients who received LMWH prophylaxis exhibited higher incidence of VTE (5.9% vs. 2.02%, *P* < 0.001), higher mortality (58.02% vs. 52.47%, *P* = 0.005), and longer length of stay compared to those who did not receive LMWH prophylaxis. No significant differences were observed between the two groups in baseline IMPROVE bleeding risk scores and bleeding rates (Table 1).

**Table 1:** Baseline characteristics and clinical outcomes of in-hospital COVID-19 patients with and without LMWH thromboprophylaxis

	No Thromboprophylaxis (*n* = 2529)	LMWH Thromboprophylaxis (*n* = 1575)	Total (*n* = 4104)	*P*
**Baseline characteristics**				
Age				< 0.0001
≤65	908 (35.90)	367 (23.27)	1275 (31.05)	
>65	1621 (64.10)	1210 (76.73)	2831 (68.95)	
Sex				< 0.0001
Male	1460 (57.73)	1033 (65.50)	2493 (60.72)	
Female	1069 (42.27)	544 (34.50)	1613 (39.28)	
BMI(Kg/m^2^)				0.3600
<18.5	162 (9.23)	82 (8.68)	244 (9.04)	
18.5–28	1443 (82.22)	767 (81.16)	2210 (81.85)	
≥28	150 (8.55)	96 (10.16)	246 (9.11)	
Smoke	280 (17.09)	224 (19.91)	504 (18.24)	0.0596
Vaccine	1060 (65.55)	502 (58.10)	1562 (62.96)	0.0003
Severity of COVID-19 at admission				< 0.0001
Moderate	1963 (77.87)	934 (59.30)	2897 (70.73)	
Severe	368 (14.60)	419 (26.60)	787 (19.21)	
Critical	190 (7.54)	222 (14.10)	412 (10.06)	
D-dimer	2.33 ± 6.79	3.00 ± 6.02	2.59 ± 6.50	0.0023
PADUA				< 0.0001
≤4	1675 (66.23)	806 (51.11)	2481 (60.42)	
>4	854 (33.77)	771 (48.89)	1625 (39.58)	
IMPROVE-DD RAM				< 0.0001
0–1	1325 (52.39)	594 (37.67)	1919 (46.74)	
2–3	904 (35.75)	706 (44.77)	1610 (39.21)	
≥4	300 (11.86)	277 (17.56)	577 (14.05)	
3D-PAST RAM				< 0.0001
<3	1257 (57.19)	552 (40.86)	1809 (50.97)	
≥3	941 (42.81)	799 (59.14)	1740 (49.03)	
IMPROVE-bleed RAM				0.2029
≤7	2295 (90.75)	1412 (89.54)	3707 (90.28)	
>7	234 (9.25)	165 (10.46)	399 (9.72)	
**Comorbidities**				
Cardiovascular disease	1452 (73.22)	1042 (77.30)	2494 (74.87)	0.0077
Respiratory disease	506 (25.56)	335 (24.87)	841 (25.28)	0.6552
Metabolic disease	773 (39.06)	570 (42.25)	1343 (40.35)	0.0652
VTE history	45 (2.24)	39 (2.87)	84 (2.49)	0.2516
Thrombophilia	3 (0.15)	1 (0.07)	4 (0.12)	0.5193
Active cancer	246 (73.00)	113 (58.55)	359 (67.74)	0.0006
**Clinical outcomes**				
VTE	51 (2.02)	93 (5.90)	144 (3.51)	< 0.0001
Death	1327 (52.47)	915 (58.02)	2242 (54.60)	0.0005
Bleeding	41 (1.62)	27 (1.71)	68 (1.66)	0.8243
Length of hospital stay	13.02 ± 9.79	15.29 ± 16.03	13.89 ± 12.60	< 0.0001

Note: VTE and mortality rates were higher in LMWH group due to bias of disease severity. Abbreviation: LMWH, low-molecular-weight heparin; BMI, body mass index; IMPROVE, the International Medical Prevention Registry on Venous Thromboembolism; RAM, risk assessment model; VTE, venous thromboembolism

### Distribution of the average dose and prophylactic duration of LMWH in diverse groups

**Fig. 2:**
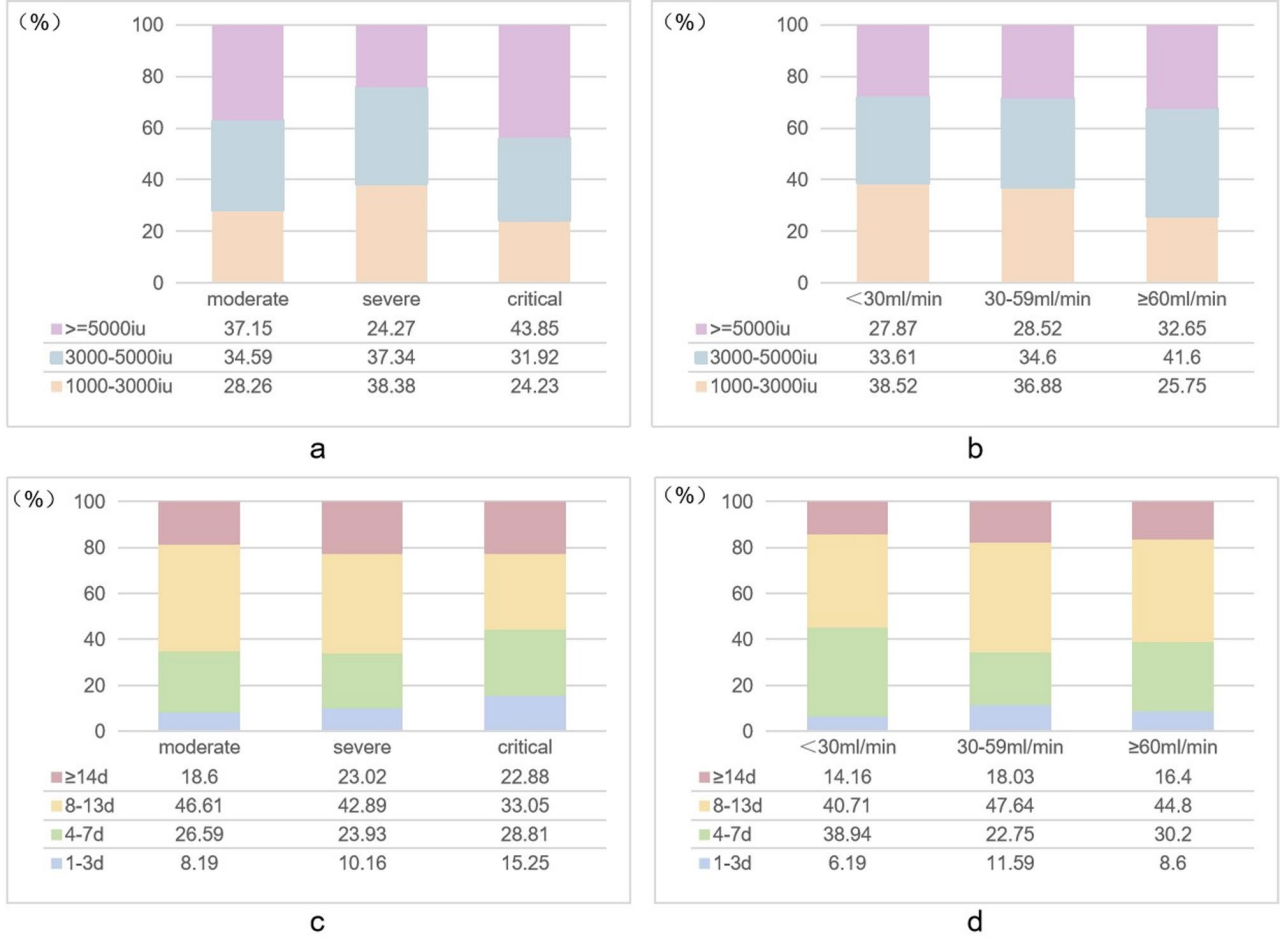
Distribution of average dose and prophylactic duration of LMWH in different subgroups stratified by disease severity and renal function Note: (**a**) Distribution of the LMWH dose in different groups of severity; (**b**) Distribution of the LMWH dose in different groups of renal function; (**c**) Distribution of prophylactic duration of LMWH in different groups of severity; (**d**) Distribution of prophylactic duration of LMWH in different groups of renal function

Overall, 592 patients received LMWH prophylaxis at reduced dosage. Among moderately and critically ill patients, 37.15% and 43.85%, respectively, received an average daily dose of at least 5,000 IU, while 38.38% of severely ill patients received 1,000–3,000 IU daily (Fig. 2a). Stratified analysis by creatinine clearance rates (CCR) revealed that patients with lower CCR were more likely to receive lower-than-conventional dosage (Fig. 2b). Further analysis of the duration of in-hospital LMWH anticoagulation indicated that a course of 8–13 days was the most common among all hospitalized COVID-19 patients (Fig. 2c and d). A higher percentage of patients with a body mass index (BMI) < 18.5 kg/m^2^ (11.05% vs. 6.92%) was observed in reduced-dose group compared to conventional-dose group. There were no significant differences in other clinical characteristics such as age, sex, D-dimer levels, comorbidities and risk scores of VTE and bleeding events at baseline (Supplementary Table 3).

### Factors influencing the choice of dosage and course of thromboprophylaxis with LMWH

Multivariable logistic regression model identified predictors of the implementation of thromboprophylaxis with LMWH during hospitalization, including age ≥ 65 (OR: 1.254; 95% CI: 1.048,1.500; *P* = 0.0134), D-dimer ≥ 0.5 mg/L at admission (OR: 2.002; 95% CI: 1.600-2.505; *P* < 0.001), severity of COVID-19 at admission (severe vs. moderate: OR: 1.566; 95% CI: 1.311–1.872; *P* < 0.001, critical vs. moderate: OR: 1.497; 95% CI: 1.183–1.895; *P* = 0.0008), and on concomitant antiviral drugs (OR: 2.164; 95% CI: 1.803,-2.596; *P* < 0.001) or corticosteroids (OR: 1.935; 95% CI: 1.674–2.236; *P* < 0.001). Patients with platelet less than 100*10^9^/L tended to not receive thromboprophylaxis with LMWH (OR: 0.584; 95% CI: 0.452–0.754; *P* < 0.001) (Table 2).

**Table 2:** Patient characteristics associated with thromboprophylaxis strategies using LMWH during hospitalization

	Implementation of LMWH					Reduced prophylactic doses					Duration of LMWH prophylaxis over 7 days				
	Univariable Analysis		Multivariable Analysis			Univariable Analysis		Multivariable Analysis			Univariable Analysis		Multivariable Analysis		
	Crude OR (95% CI)	*P*	Adjusted OR (95% CI)		*P*	Crude OR (95% CI)	*P*		Adjusted OR (95% CI)	*P*	Crude OR (95% CI)	*P*		Adjusted OR (95% CI)	*P*
**Female (vs. male)**	0.736(0.647,0.837)	< 0.0001	0.838(0.723,0.970)	0.0183		0.987(0.797,1.224)	0.9064				0.936(0.751,1.166)	0.5563			
**Age ≥ 65**	1.778(1.544,2.048)	< 0.0001	1.254(1.048,1.500)	0.0134		0.929(0.729,1.184)	0.5521				1.080(0.844,1.381)	0.5419			
**Cardiovascular disease**	1.396(1.227,1.589)	< 0.0001				0.880(0.708,1.093)	0.2461				0.961(0.770,1.200)	0.7263			
**Respiratory disease**	1.068(0.916,1.245)	0.4011				0.923(0.720,1.184)	0.529				1.003(0.776,1.297)	0.98			
**Metabolic disease**	1.263(1.107,1.441)	0.0005				1.125(0.908,1.393)	0.281				1.625(0.763,3.457)	0.2079			
**Active cancer**	0.731(0.580,0.921)	0.0078				1.105(0.741,1.649)	0.6231				0.904(0.606,1.350)	0.6219			
PLT(*10 ^**9**^ **/L)**															
≥200	1.000[Ref]	-	1.000[Ref]	-							1.000[Ref]			1.000[Ref]	
100–200	0.970(0.849,1.108)	0.656	0.863(0.742,1.003)	0.0544		0.845(0.680,1.051)	0.131		0.827(0.652,1.050)	0.1189	0.590(0.401,0.868)	0.5022		0.914(0.717,1.167)	0.472
< 100	0.723(0.575,0.910)	0.0057	0.584(0.452,0.754)	< 0.0001		0.835(0.565,1.233)	0.3643		0.735(0.483,1.120)	0.1523	0.926(0.739,1.159)	0.0074		0.551(0.363,0.836)	0.0051
**Anemia**	1.133(0.989,1.298)	0.0711	0.943(0.803,1.107)	0.4745							0.790(0.628,0.995)	0.0452		0.723(0.565,0.924)	0.0097
**D-dimer(mg/L)**															
< 0.5	1.000[Ref]	-	1.000[Ref]	-		1.000[Ref]	-		1.000[Ref]	-	1.000[Ref]	-			
≥ 0.5	2.374(1.947,2.895)	< 0.0001	2.002(1.600,2.505)	< 0.0001		0.806(0.386,1.311)	0.3659		0.911(0.404,1.355)	0.4206	0.854(0.605,1.205)	0.3678			
**eGFR(mL/min/1.73 m** ^**2**^ **)**															
≥90	1.000[Ref]	-	1.000[Ref]			1.000[Ref]			1.000[Ref]		1.000[Ref]	-		1.000[Ref]	-
60–90	1.497(1.292,1.734)	< 0.0001	1.145(0.956,1.373)	0.1418		0.779(0.606,1.001)	0.0513		0.895(0.666,1.203)	0.7314	1.282(0.997,1.649)	0.053		1.483(1.106,1.989)	0.0085
< 60	1.680(1.389,2.032)	< 0.0001	1.236(0.980,1.558)	0.0735		0.831(0.607,1.138)	0.2495		0.938(0.652,1.350)	0.4621	1.556(1.122,2.157)	0.008		1.731(1.191,2.518)	0.0041
**Severity of COVID-19 at admission**															
Moderate	1.000[Ref]	-	1.000[Ref]	-		1.000[Ref]	-		1.000[Ref]	-	1.000[Ref]	-		1.000[Ref]	-
Severe	2.214(1.893,2.588)	< 0.0001	1.566(1.311,1.872)	< 0.0001		0.714(0.565,0.904)	0.005		0.703(0.541,0.912)	0.008	1.083(0.845,1.388)	0.5276		0.978(0.743,1.287)	0.8745
Critical	2.207(1.804,2.701)	< 0.0001	1.497(1.183,1.895)	0.0008		1.329(0.968,1.826)	0.079		1.535(1.071,2.201)	0.0197	0.736(0.544,0.996)	0.0471		0.747(0.528,1.056)	0.0987
**Antiviral drugs**	2.340(1.991,2.751)	< 0.0001	2.164(1.803,2.596)	< 0.0001		0.983(0.777,1.245)	0.889		1.031(0.794,1.339)	0.8163	1.515(1.176,1.952)	0.0013		1.389(1.056,1.829)	0.019
**Corticosteroids**	2.309(2.033,2.622)	< 0.0001	1.935(1.674,2.236)	< 0.0001		0.938(0.761,1.157)	0.5512		0.917(0.722,1.164)	0.4758	1.273(1.029,1.577)	0.0265		1.371(1.078,1.745)	0.0102

Abbreviation: LMWH, low-molecular-weight heparin; PLT, platelet; eGFR, estimated glomerular filtration rate; OR, odds ratio; CI, confidence intervals

As for reduced-dosage prophylactic LMWH, critically ill patients at admission were more likely to receive reduced prophylactic doses of LMWH (critical vs. moderate: OR: 1.535; 95% CI: 1.071–2.201; *P* = 0.0197), while severely ill at admission was associated with conventional-dose prophylaxis (severe vs. moderate: OR: 0.703; 95% CI: 0.541–0.912; *P* = 0.008) (Table 2).

Factors associated with LMWH prophylaxis lasting over 7 days included estimated glomerular filtration rate (eGFR) < 60mL/min/1.73m^2^ (vs. eGFR ≥ 90 mL/min/1.73 m: OR: 1.731; 95% CI: 1.191–2.518; *P* = 0.0041), eGFR 60-90mL/min/1.73m^2^ (vs. eGFR ≥ 90 mL/min/1.73 m: OR: 1.483; 95% CI: 1.106–1.989; *P* = 0.0085), and on concomitant antiviral drugs (OR: 1.389; 95% CI: 1.056–1.829; *P* = 0.019) or corticosteroids (OR: 1.371; 95% CI: 1.078–1.745; *P* = 0.0102). Patients with platelets less than 100*10^9^/L (OR: 0.551; 95% CI: 0.363–0.836; *P* = 0.0051), anemia (OR: 0.723; 95% CI: 0.565–0.924; *P* = 0.0097) tended to receive thromboprophylaxis with LMWH less than 7 days (Table 2). While there was no significant difference in VTE incidence, mortality, or bleeding rates between the low-dose and conventional-dose groups, patients receiving reduced prophylactic doses had a shorter hospital stay (14.01 ± 13.40 days vs. 15.77 ± 16.69 days, *p* = 0.0203) (Supplementary Table 3).

## Discussion

The exploration of VTE prevention strategies in China is critically relevant, particularly in the aftermath of the COVID-19 epidemic. In the current study, we found that 37.09% of the in-hospital COVID-19 patients received LMWH prophylaxis, in which over one third received reduced dosage without increasing VTE incidence or mortality. Physicians tended to use reduced prophylactic dose in patients who were critically ill at admission. Anemia and platelet less than 100*10^9^/L might be the important characteristic for physicians in deciding LMWH prophylactic course.

Optimal thromboprophylaxis strategies remain controversial. Current guidelines recommend prophylactic doses for ICU-admitted COVID-19 patients and therapeutic doses for non-ICU patients [11, 12]. It is of particular concern that there has been an increased incidence of major bleeding among critically ill patients receiving therapeutic anticoagulation for prophylaxis [14]. Furthermore, east Asian populations may have a lower risk of VTE compared to western populations, potentially due to genetic predispositions and lifestyle factors [21]. Studies have reported that East Asians tend to exhibit lower level of lipoprotein (a) [22], reduced platelet–fibrin clot strength measured by thromboelastography [23], and decreased inflammation indicated by C-reactive protein [24], relative to other races. These differences may influence the physiological response to anticoagulation and complicate treatment optimization in this population. Research from Japan has revealed inconsistencies in thromboprophylaxis with unfractionated heparin in hospitalized COVID-19 patients between the real clinical practices and guidelines, highlighting the necessity for region-specific recommendations [25]. However, they did not evaluate the detailed dosage and duration of pharmacological thromboprophylaxis.

Our findings showed that age over 65 years, elevated D-dimer levels at admission, and the severity of COVID-19 were predictors of the implementation of LMWH thromboprophylaxis during hospitalization, which was thought to be an important characteristic for physicians in deciding management strategies. These factors were aligned with the risk factors of VTE identified in prior research [26, 27]. Additionally, the use of concomitant antiviral drugs and corticosteroids, which can modulate the inflammatory and immune response, also had an influence on the clinician’s decision of thromboprophylaxis. Corticosteroids may concurrently elevate bleeding risks [28], however, the impact of antiviral drugs and corticosteroids on the thrombotic risk remains uncertain. Further investigations are essential to explore the complex interactions between different drugs and their implications for thrombotic risk management in COVID-19 patients.

The choice of LMWH dosage is critical and often adjusted based on patient-specific factors. Critically ill patients were more likely to receive reduced prophylactic doses, which is in accordance with findings from some randomized controlled trials. Patients who need ICU-level care, defined as the use of respiratory or cardiovascular organ support, exhibited higher bleeding rate without a significant reduction in thrombosis after receiving intermediate- or therapeutic-dose anticoagulation [29–31]. According to autopsy research, it was possible that marked pulmonary inflammation may exacerbate alveolar hemorrhage with therapeutic-dose anticoagulation, leading to worse outcomes [32]. Additionally, prior clinical experience, evolving evidence during the pandemic, and cautious judgment in managing patients with multi-organ dysfunction may lead to more conservative dosing in real-world settings. These findings suggest that reducing anticoagulation doses in certain populations may be beneficial.

Currently, there is a lack of evidence regarding the optimal duration of prophylactic anticoagulation therapy for COVID-19 patients. Our results found the duration of LMWH prophylaxis varied, with a course of 7–14 days being the most common. The distribution of length of hospital stay is located within 5 days to 21 days. Patients with thrombocytopenia or anemia frequently received shorter anticoagulation courses. This may reflect the needs to balance the thrombotic benefits with potential risks of prolonged anticoagulation, such as bleeding or heparin-induced thrombocytopenia [33]. Patients with renal dysfunction have a significantly higher risk of thrombosis due to endothelial dysfunction, accumulation of inflammatory factors, and immune disorders, which are further exacerbated by coagulation abnormalities after infection [34]. Therapeutic doses of anticoagulation may increase the incidence of acute kidney injury, while prophylactic doses are relatively safe [35]. For patients with impaired renal function, doctors may prefer to extend prophylactic anticoagulation rather than escalate to therapeutic doses. Corticosteroids or antiviral drugs are frequently employed in the treatment of severe COVID-19 patients, who are already at elevated risk of thrombosis. These patients often require prolonged anticoagulation prophylaxis.

The study was subject to some limitations. First, given the observational design and the complexity of clinical decision-making, our findings cannot directly inform how LMWH dosage or duration should be adjusted. However, regarding the inclusion of patients from various regions across China, our cohort demonstrates good national representativeness of describing the real-world patterns of pharmacological prophylaxis of VTE in Chinese hospitalized COVID-19 patients. Second, the study did not account for transitions between different anticoagulants, limited to the comprehensiveness of the clinical data. Lastly, our study included only patients infected during the Omicron-dominant phase of the COVID-19 pandemic. As pathogenicity can vary across SARS-CoV-2 variants, the findings may not be fully generalizable to other periods dominated by different variants such as Delta or the original strain.

However, the strengths of our study should also be acknowledged. Our study provided a comprehensive and detailed analysis of current thromboprophylaxis with LMWH in in-hospital COVID-19 patients, revealing gaps between the clinical practice and the recommendations from guidelines. Furthermore, we identified the underrepresented populations with a lack of evidence in thromboprophylaxis, indicating the need for further exploration.

Future research should include randomized controlled trials to evaluate the efficacy and safety of different LMWH doses and durations in subgroups stratified by disease severity and comorbidities, particularly in fragile populations and those with complex comorbid conditions. While our study focuses on COVID-19, the insights into individualized anticoagulation strategies may be relevant to other infectious associated with elevated VTE risk, such as influenza, other viral infections or bacterial sepsis. Further studies are needed to validate the applicability of these strategies beyond COVID-19, particularly in populations with similar inflammatory profiles.

## Conclusion

Real-world thromboprophylaxis in hospitalized COVID-19 patients vary widely, with a significant proportion receiving reduced doses of LMWH. There is a need for personalized thromboprophylaxis strategies that consider patient-specific factors such as disease severity, renal function, bleeding risk and concomitant medications to optimize outcomes.

## Electronic supplementary material

Below is the link to the electronic supplementary material.

Supplementary Material 1

Supplementary Material 2

## Data Availability

The data that support the findings of this study are not openly available due to reasons of sensitivity and are available from the corresponding author upon reasonable request.

## Abbreviations

BMI: Body mass index
CCR: Creatinine clearance rates
Cis: Confidence intervals
CRNMB: Clinically relevant non-major bleeding
DVT: Deep vein thrombosis
eCRF: Electronic reporting form
eGFR: Estimated glomerular filtration rate
ICU: Intensive care unit
IMPROVE: The International Medical Prevention Registry on Venous Thromboembolism
ISTH: International Society of Thrombosis and Hemostasis
LMWH: Low-molecular-weight heparin
MR: Major bleeding
OR: Odds ratio
PE: Pulmonary embolism
RAM: Risk assessment model
VTE: Venous thromboembolism
