# Supplementary material for: Real-world practices of low-molecular-weight heparin for venous thromboembolism prophylaxis in patients hospitalized with COVID-19: a multicenter prospective study from China
Source: Thromb J. 2025 Jun 20;23:69. doi: 10.1186/s12959-025-00741-9 (PMC12181860; doi:10.1186/s12959-025-00741-9)
Supplement: Supplementary file 2 — Supplementary Material 2 [file 12959_2025_741_MOESM2_ESM.docx]

**Supplementary table 1. Geographical distribution of enrolled participants across China**

| Province of China | Patients |
| --- | --- |
| Yunnan Province | 210 |
| Zhejiang Province | 320 |
| Ningxia Province | 958 |
| Guizhou Province | 894 |
| Fujian Province | 748 |
| Beijing | 1106 |

**Supplementary table 2.** **Distribution of other anticoagulants in VTE prevention**

| Anticoagulants | n (%) |
| --- | --- |
| Unfractionated heparin | 52 (1.22) |
| Fondaparinux Sodium | 12 (0.28) |
| Argatroban | 0 |
| NOACs (Rivaroxaban, Pradaxa, Apixaban, Edoxaban) | 284(6.68) |
| Warfarin | 26 (0.61) |

Abbreviation: VTE, venous thromboembolism; NOACs, Novel Oral Anticoagulants drugs.

**Supplementary table 3. Baseline characteristics and clinical outcomes of in-hospital COVID-19 patients with conventional and reduced prophylactic doses of LMWH**

|  | **Reduced prophylactic doses (n=592)** | **Conventional prophylactic doses (n=983)** | **P** |
| --- | --- | --- | --- |
| Age |  |  | 0.5521 |
| ≤65 | 385 (65.25) | 643 (65.55) |  |
| >65 | 205 (34.75) | 338 (34.45) |  |
| Sex |  |  | 0.9065 |
| Male | 385 (65.25) | 643 (65.55) |  |
| Female | 205 (34.75) | 338 (34.45) |  |
| BMI (kg/m^2^) |  |  | 0.0519 |
| <18.5 | 40 (11.05) | 40 (6.92) |  |
| 18.5-28 | 291 (80.39) | 474 (82.01) |  |
| ≥28 | 31 (8.56) | 64 (11.07) |  |
| Vaccine | 182 (30.85) | 319 (32.52) | 0.4915 |
| Severity of COVID-19 at admission | |  | <.0001 |
| Moderate | 339 (57.46) | 595 (60.65) |  |
| Severe | 185 (31.36) | 232 (23.65) |  |
| Critical | 66 (11.19) | 154 (15.70) |  |
| D-dimer (mg/L) | 1.24 (0.74, 2.30) | 1.23 (0.63, 2.64) | 0.2938 |
| PADUA |  |  | 0.5985 |
| ≤4 | 307 (52.03) | 497 (50.66) |  |
| >4 | 283 (47.97) | 484 (49.34) |  |
| IMPROVE_DD RAM | |  | 0.1102 |
| 0-1 | 228 (38.64) | 364 (37.10) |  |
| 2-3 | 274 (46.44) | 430 (43.83) |  |
| ≥4 | 88 (14.92) | 187 (19.06) |  |
| 3D-PAST RAM |  |  | 0.3609 |
| <3 | 205 (42.53) | 345 (39.98) |  |
| ≥3 | 277 (57.47) | 518 (60.02) |  |
| IMPROVE-bleed RAM | |  | 0.2243 |
| ≤7 | 519 (87.67) | 879 (89.43) |  |
| >7 | 73 (12.33) | 104 (10.57) |  |
| **Comorbidities** |  |  |  |
| Cardiovascular disease | 400 (67.80) | 637 (64.93) | 0.2460 |
| Respiratory disease | 130 (22.03) | 203 (20.69) | 0.5289 |
| Metabolic disease | 203 (34.41) | 364 (37.10) | 0.2809 |
| VTE history | 14 (2.37) | 24 (2.45) | 0.9267 |
| Thrombophilia | 1 (0.1965) | 1 (0.1965) | 0.3773 |
| Active cancer | 40 (6.78) | 73 (7.44) | 0.6230 |
| **Clinical outcome** |  |  |  |
| VTE | 31 (5.25) | 60 (6.12) | 0.4788 |
| Death | 122 (20.68) | 207 (21.10) | 0.8419 |
| Bleed | 13 (2.36) | 21 (2.20) | 0.8174 |
| Length of hospital stay | 14.01 ± 13.40 | 15.77 ± 16.69 | 0.0203 |

Abbreviation: LMWH, low-molecular-weight heparin; BMI, body mass index; VTE, venous thromboembolism; IMPROVE, the International Medical Prevention Registry on Venous Thromboembolism; RAM, risk assessment model.
